# A quasi-experimental study on the impact of interprofessional education on collaborative attitudes among midwifery, nursing, and medicine students in Brussels, Belgium

**DOI:** 10.18332/ejm/204273

**Published:** 2025-07-03

**Authors:** Joeri Vermeulen, Ronald Buyl, Ives Hubloue, Sofie Pauwels, Marc Diltoer, Lara Stas, Merjem Ouelhadj, Elke Moortgat, Maaike Fobelets

**Affiliations:** 1 Faculty of Medicine and Pharmacy, Department of Public Health, Biostatistics and Medical Informatics Research Group, Vrije Universiteit Brussel, Brussels, Belgium; 2 Department of Life Sciences and Medicine, Faculty of Science, Technology and Medicine, University of Luxembourg, Eschsur-Alzette, Luxembourg; 3 Department of Health Care, Erasmus Brussels University of Applied Sciences and Arts, Brussels, Belgium; 4 Department of Emergency Medicine, University Hospital Brussels, Brussels, Belgium; 5 Faculty of Medicine and Pharmacy, ReGEDiM Research Group on Emergency and Disaster Medicine, Vrije Universiteit Brussel, Brussels, Belgium; 6 Intensive Care Unit, University Hospital Brussels, Brussels, Belgium; 7 Faculty of Medicine and Pharmacy, Vrije Universiteit Brussel, Brussels, Belgium; 8 Brussels Institute for Teacher Education, Vrije Universiteit Brussel, Brussels, Belgium

**Keywords:** healthcare students, midwifery, medicine, nursing, interprofessional education, interprofessional collaboration

## Abstract

**INTRODUCTION:**

Interprofessional education (IPE) positively influences students’ attitudes toward interprofessional collaboration, as demonstrated by multiple studies. However, few studies have examined IPE effects across more than two disciplines. Further investigation is needed to assess its impact on students’ attitudes across diverse healthcare fields. This study aimed to evaluate shifts in students’ attitudes toward interprofessional collaboration and their perceptions of the intervention’s educational value and satisfaction.

**METHODS:**

The IPE intervention took place at Erasmus Brussels University of Applied Sciences in Brussels, Belgium between 2021 and 2022. Final-year nursing and midwifery students, along with fourth-year medical students, were randomly assigned to heterogeneous teams to address topics such as Basic and Advanced Life Support. The study used a quasi-experimental design with pre- and post-test evaluations, employing the Interprofessional Education Perception Scale (IEPS) to measure attitude shifts. Additionally, qualitative assessment was conducted using an open-ended question.

**RESULTS:**

A total of 269 healthcare students participated. Significant improvements in competence, autonomy, and perception of collaboration were found post-intervention (p<0.001). Positive attitude shifts were consistent across age, gender, and educational backgrounds. Qualitative data highlighted strong student support for the intervention and recognition of its educational value.

**CONCLUSIONS:**

This study underscores the need for structured IPE pathways and early integration into healthcare curricula. It also highlights existing gaps in IPE and offers recommendations for enhancing midwifery education and practice.

## INTRODUCTION

According to the World Health Organization, interprofessional education (IPE) involves students from multiple healthcare disciplines learning together to foster collaboration and improve health outcomes. It provides a safe, supportive environment for shared learning experiences^1^. Collaborative cross-professional learning contributes to the development of knowledge^2^ and competences among healthcare students^3^ thereby strengthening their capacity for interprofessional collaboration as essential contributors within multidisciplinary teams^4^. IPE additionally yields positive effects on various dimensions, such as interprofessional communication, and the cultivation of professional relationships^5^.

Research shows that IPE cultivates positive attitudes toward collaboration, strengthens clinical decision-making, and improves teamwork among healthcare professionals. By bringing together diverse disciplines, IPE enhances knowledge, competence, and interdisciplinary cooperation^3^. Both doctors and midwives have expressed appreciation for IPE’s role in promoting teamwork, communication, and mutual respect across professions^5^. Simulation-based IPE, such as Advanced Life Support and emergency medicine scenarios, has been shown to increase participants’ confidence, role understanding, and clinical performance compared to traditional training methods. Additionally, a recent meta-analysis suggested that the overall impact of IPE on healthcare students’ readiness for interprofessional collaboration remains inconclusive, with mixed findings on its long-term effects on attitudes and competencies^5^.

While numerous studies highlight the benefits of IPE, most focus on collaboration between two disciplines, leaving a gap in evaluating more diverse interprofessional teams. Though IPE improves communication and fosters appreciation for different professional roles, its broader impact on multidisciplinary collaboration and students’ attitudes toward interprofessional competencies remains underexplored^6^. Further research is needed to address these gaps, informing educational practices and policies to enhance interprofessional collaboration and ultimately improve patient outcomes. The importance of future research in this area has been emphasized, as evidenced by a recent systematic review and meta-analysis. Factors impacting students’ attitudes and readiness for interprofessional learning should be explored^2^. Consequently, this study aimed to evaluate shifts in students’ attitudes toward interprofessional collaboration and assess their perceptions of the intervention’s educational value and overall satisfaction.

## METHODS

### Study design and setting

In 2016, a collaborative IPE model was developed by a multidisciplinary team from the Vrije Universiteit Brussel (VUB), Erasmus Brussels University of Applied Sciences, and the University Hospital Brussels in Brussels, Belgium. This model was introduced within final-year nursing and midwifery students and fourth-year medical students. Midwifery, nursing, and medicine students often work closely together in healthcare settings, making their inclusion in the IPE model essential. Additionally, as there have already been established partnerships between the involved institutions and programs of midwifery, nursing, and medicine, it is a natural choice to include students from these disciplines. In the collaborative interprofessional learning intervention, students of different healthcare education programs are randomly assigned to diverse teams for half a day. Each team consists of a maximum of five members, including one midwifery student, two nursing students and two medical students. Participation is mandatory, and all scenarios are tailored to address realistic and authentic problems, aligned with the problem-oriented learning approach inherent to IPE. Key topics include Basic Life Support, Advanced Life Support, Airway, Breathing, Circulation, Disability, Exposure, and Neonatal Life Support. Each session lasts 40 minutes and is followed by a 10-min facilitated debriefing. After completion of a session, students rotate to another scenario. All students are involved in four scenario-structured simulation sessions. Each of the four sessions start with an initial orientation on the environment and continues with a simulation session, using medium and high-fidelity manikins. Medium-fidelity human patient simulation manikins are full-body manikins with embedded software^7^. They are controlled by an external, hand-held device and have limited physiological responses. High-fidelity human patient simulation manikins are defined as ‘life-like’ manikins with embedded software that can be remotely controlled by computer to allow for individualized, programmed scenarios that allow the operator to set physiological parameters and respond to participants’ interventions^8^. All students are involved in all simulation sessions, both actively and as observer. The sessions are supervised by senior midwives, nurses, doctors and educators from the Vrije Universiteit Brussel (VUB), Erasmus Brussels University of Applied Sciences, and the University Hospital Brussels in Brussels, Belgium. Students are prepared by interactive sessions covering the fundamental principles of Basic Life Support, Advanced Life Support, Airway, Breathing, Circulation, Disability, Exposure, and Neonatal Life Support. Complemented with immediate practice by all students.

### Participants

A convenience sample was used, consisting of final-year nursing and midwifery students and fourth-year medical students. The IPE sessions took place in the first semester, spanning four days with 32 groups (eight per day) at Erasmus Brussels University of Applied Sciences in Brussels, Belgium. Participants were invited and provided with information via email two weeks before the scheduled sessions (November 2021 and 2022). Before the preparatory plenary lecture, the aim of the study was explained by a staff member not involved in the simulation sessions. Consequently, informed consent was collected from participants willing to participate. Students were explicitly given the option whether they want to participate or not without any coercion or pressure. The surveys were offered to students immediately before the training and immediately after, both on the same day. All data were securely stored in an onsite locked facility, only accessible to the principal researcher (JV). No data were shared or discussed with other colleagues, while identifying information was removed, to maintain anonymity, by the principal researcher.

### Variables

The primary outcome was changes in students’ attitudes toward interprofessional collaboration (IEPS). Secondary outcomes included perceived educational value and satisfaction with the intervention. The exposure was participation in the IPE intervention. Predictors included demographics, disciplinary background, and baseline attitudes. Confounders were prior training and curriculum variability, while effect modifiers included clinical experience and disciplinary differences in IPE perceptions.

### Intervention

The IPE sessions involved a series of four simulation sessions. Each session began with an initial orientation followed by a simulation using medium- and high-fidelity manikins. All students were actively involved in the simulations or participated as observers. Following each 40-min session, a 10-min facilitated debriefing was conducted, after which students rotated to a new scenario. This intervention provided experiential learning opportunities through realistic case scenarios.

### Measurement

In subsequent years (2021 and 2022), an anonymous online survey was conducted using Qualtrics^™^. The quasi-experimental study involved pre- and post-test evaluations of students’ attitudes regarding interprofessional collaboration using the IPE Perception Scale, IEPS^9^. The survey instrument employed was the Dutch version of the IEPS, which was used in an earlier study^4^. This version of the IEPS consists of 18 statements rated on a 6-point Likert scale. Students were invited to rate statements related to their attitude towards interprofessional collaboration from strongly disagree to strongly agree. The Dutch version of the IEPS encompasses four subscales, including: 1) Competence and autonomy, 2) Perceived need for cooperation, 3) Perception of actual cooperation, and 4) Perception status of own profession. Additionally sociodemographic data were gathered, and we added one open question namely, ‘Do you have any additional comments/suggestions regarding IPE?’.

### Statistical analysis

*Reliability and validity of the Dutch version of the IEPS*

First, the reliability of each subscale of the Dutch version of the IEPS was examined. Cronbach’s alpha was used as a measure of internal consistency, the alpha should be at least 0.70 to consider the internal consistency of the subscale acceptable. From 0.80 onwards, a subscale has a good internal consistency. The subscale ‘Competence and autonomy’ obtained a Cronbach’s alpha of 0.84, while the subscale ‘Perception of actual cooperation’ received 0.81, and are both considered internally consistent. The subscales ‘Perceived need for cooperation’ (which consists of only two items, leading Cronbach’s alpha to potentially underestimate the true reliability) and ‘Perception status of own profession’ (with a Cronbach’s alpha of 0.51) were found to lack internal consistency and, as a result, were excluded from subsequent analyses.

Next, validity checks of the proposed scales were performed. For this purpose, confirmatory factor analysis (CFA) models were constructed, using the R package *lavaan*. Supplementary file Section 1 contains more details on the model building procedure and validity checks^10^. The final model was estimated using diagonally weighted least squares estimator and contains two subscales: ‘Competence and autonomy’ (7 items) and ‘Perception of actual cooperation’ (4 items). Overall, this final model appears to have good construct and convergent validity. The model fit indices show an excellent model fit ($\chi_{43}^{2}$ =46.087, p=0.346; Comparative Fit Index = 0.998, Tucker-Lewis Index = 0.997, RMSEA = 0.020). The factor loadings for the items on their respective factors are all significant and moderate to high, ranging from 0.493 to 0.775. This suggests that the factors are related to their respective items and that they are measuring the intended construct. Regarding discriminant validity, we found a correlation between the latent factors ‘Competence and autonomy’ and ‘Perception of actual cooperation’ of 0.834 (p<0.001). In conclusion, the revised Dutch version of the IEPS is a reliable and valid tool for measuring ‘Competence and autonomy’ and ‘Perception of actual cooperation’ (see Supplementary file Section 2).

*Quantitative and qualitative analysis of the data*

The data were described using frequencies (n) and percentages (%) for discrete outcome variables, and mean ± standard deviation (SD) for continuous outcomes, broken down by gender, educational program and age categories. Differences in pre-test/post-test changes between respectively the medical, nursing and midwifery students were calculated using a paired samples t-test. All statistical analysis were performed using the SPSS software Statistics for Windows, version 28.0, a p≤0.05 was considered significant.

To analyze the responses to the query concerning additional remarks and recommendations regarding IPE, a qualitative content analysis approach has been employed. Additionally, direct quotations from respondents were included to provide specific examples of their feedback. This qualitative approach allowed for a rich understanding of participants’ perspectives on IPE.

## RESULTS

Healthcare students (n=269) from three distinct educational programs, midwifery, nursing, and medicine participated, comprising 217 females and 52 males. Specifically, the medicine program enrolled 117 participants, the nursing program had a total of 77 participants, and the midwifery program included 75 students (Table 1).

**Table 1:** Participants distribution across three educational programs

	*Educational program, n (%)*			
	*Medicine*	*Nursing*	*Midwifery*	*Total*
**Female**	80 (68.38)	63 (81.82)	74 (98.67)	217 (80.67)
**Male**	37 (31.62)	14 (18.18)	1 (1.33)	52 (19.33)
**Total**	117 (100)	77 (100)	75 (100)	269 (100)

The participation rates for three distinct groups in a study, as shown in Table 2, revealed varying levels of engagement throughout the research phases. Initially, all groups demonstrated high pre-test participation rates (range: 92.5–97.5%). However, during the post-test phase, participation rates declined across the board. Medicine students retained the highest post-test participation rate at 72.6%, while nursing students experienced a substantial drop to 37.6%, and the midwifery students exhibited a decline to 61.3%.

**Table 2:** Pre- and post-test participation rate

	*Participation rate, n (%)*		
	*Medicine*	*Nursing*	*Midwifery*
**Participation pre-test**	117 (97.5)	77 (93.9)	75 (92.5)
**Participation post-test**	85 (72.6)	29 (37.6)	46 (61.3)

The results of the paired sample t-test analysis suggest that both ‘Competence and autonomy’, as well as ‘Perception of actual collaboration’, exhibited statistically significant improvements from the pre-test to the post-test (both p<0.001). The positive mean differences indicate that, on average, participants scored higher in both variables after the intervention or program among respective medicine, nursing, and midwifery students. Importantly, this increase in positive attitude was consistent across age, gender, and educational backgrounds.

Table 3 presents a comprehensive analysis of how responses to competency and autonomy-related survey statements, such as ‘People from my professional group demonstrate great autonomy’ and ‘People from my professional group trust each other’s professional judgment’, evolved before and after the IPE intervention. While analyzing the percentage of students who ‘strongly agree’ with competency and autonomy-related statements, the highest increase is observed in midwifery students (+10.2% post-test), followed closely by medicine students with a significant increase of 9.0% post-test, and the lowest increase is seen in nursing students (+5.3% post-test). In addition, Table 3 provides an analysis of how responses to perception of actual collaboration among medicine, nursing, and midwifery students, as reflected in survey statements such as ‘People from my professional group are capable of collaborating closely with people from other professional groups’ and ‘People from my professional group have good relationships with people in other professional groups’, evolved before and after the IPE intervention. Similarly, as with the competency and autonomy-related statements, of the students who strongly agreed with actual collaboration-related statements, we noted the highest increase among midwifery students (+27.6% post-test), followed by medical students with an increase 25.8% post-test, and the lowest increase among nursing students (+6.7% post-test).

**Table 3:** Differences in competence, autonomy and perception of actual collaboration

Differences in competence and autonomy (%)																	
*Medicine pre-test (N=117)*						*Nursing pre-test (N=77)*						*Midwifery pre-test (N=75)*					
0	1	2	3	4	5	0	1	2	3	4	5	0	1	2	3	4	5
1.1	5.8	0.4	41.1	20.6	31.1	1.5	3.6	1.3	34.3	23.7	33.5	0.5	8.0	2.6	42.0	23.0	27.5
***Medicine post-test (N=85)***						***Nursing post-test (N=29)***						***Midwifery post-test (N=46)***					
0	1	2	3	4	5	0	1	2	3	4	5	0	1	2	3	4	5
1.3	1.0	1.2	40.6	12.5	40.1	0.4	1.5	1.5	39.6	17.6	38.8	1.6	5.9	2.4	38.9	14.9	37.7
***Differences in actual collaboration (%)***																	
***Medicine pre-test (N=117)***						***Nursing pre-test (N=77)***						***Midwifery pre-test (N=75)***					
0	1	2	3	4	5	0	1	2	3	4	5	0	1	2	3	4	5
0.67	2.8	1.1	44.3	30.5	20.7	2.3	1.0	0.7	35.8	18.9	41.6	1.7	4.7	0.0	41.7	22.0	30.0
***Medicine post-test (N=85)***						***Nursing post-test (N=29)***						***Midwifery post-test (N=46)***					
0	1	2	3	4	5	0	1	2	3	4	5	0	1	2	3	4	5
1.2	1.0	2.7	38.8	10.0	46.5	0.4	0.9	0.9	33.5	16.1	48.3	1.1	0.0	2.7	31.5	7.1	57.6

Scale: 0=strongly disagree, 1=somewhat disagree, 2=slightly disagree, 3=slightly agree, 4=somewhat agree, and 5=strongly agree.

**Figure 1:**
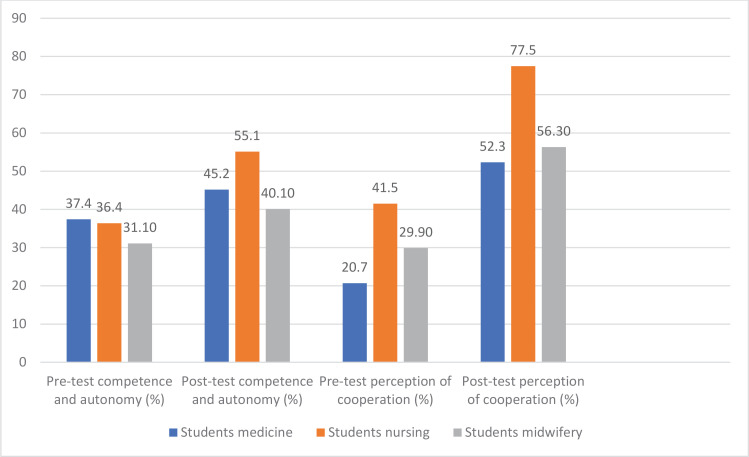
Competence and autonomy, and perception of cooperation: pre- and post-test

Table 4 and Figure 1 present data related to the perceptions and attitudes of medicine, nursing, and midwifery students, before and after the IPE intervention. Overall, the data are presented in percentages of students who ‘strongly agree’ with various statements related to competence, autonomy, and collaboration within their respective professional groups. The findings of the present study demonstrate that the IPE intervention positively impacted the students’ attitudes and perceptions, the percentage of students who strongly agree with statements related to competence and autonomy and actual collaboration increased for students after the intervention. In the ‘Competence and Autonomy’ dimension, there was a decrease observed after the intervention in one item, specifically among medicine students, with the item ‘People from my professional group are highly competent’ showing a pre-test percentage of 23.0% compared to 19.8% post-test. Notably, the highest post-test scores for all three student groups were found for the item ‘People from my professional group need to collaborate with other professional groups’, with medicine students at 94.2%, nursing students at 75.8%, and midwifery students at 69.5%. In the ‘Actual Collaboration’ dimension, the highest post-test scores were observed in medicine (59.7%) and midwifery (63.0%) students for the item ‘People from my professional group are willing to share information and resources with other professional groups’. Similarly, nursing students achieved the highest post-test score (96.5%) for the item ‘People from my professional group work well together’, which was also a shared highest score for midwifery students (63.0%).

**Table 4:** Differences in competence and autonomy, and perception of actual cooperation across three educational programs

	*Medicine students* *(% strongly agree)*		*Nursing students* *(% strongly agree)*		*Midwifery students* *(% strongly agree)*	
	*Pre* *(N=117)*	*Post* *(N=85)*	*Pre* *(N=77)*	*Post* *(N=29)*	*Pre* *(N=75)*	*Post* *(N=46)*
**Competence and autonomy**	37.4	45.2	36.4	55.1	31.1	40.1
People from my professional group demonstrate great autonomy	22.2	26.5	32.4	27.5	16.0	21.7
People from other professional groups respect the work performed by my professional group	38.4	71.7	29.8	65.5	24.0	50.0
People from my professional group have a very positive attitude toward their own goals and objectives	31.6	38.4	29.8	51.7	22.6	41.3
People from my professional group need to collaborate with other professional groups	82.9	94.2	64.9	75.8	52.0	69.5
People from my professional group are very positive about their contributions and achievements	29.0	34.5	33.7	58.6	30.6	28.2
People from my professional group trust each other’s professional judgment	24.7	46.4	38.9	62.0	29.3	45.6
People from my professional group are highly competent	23.0	19.8	28.5	55.1	18.6	23.9
**Perception of actual cooperation**	20.7	52.3	41.5	77.5	29.9	56.3
People from my professional group can collaborate closely with people from other professional groups	20.5	55.7	46.7	79.3	26.6	54.3
People from my professional group are willing to share information and resources with other professional groups	31.6	59.7	50.6	72.4	34.6	63.0
People from my professional group have good relationships with people in other professional groups	11.1	45.1	31.1	62.0	25.3	45.0
People from my professional group work well together	19.6	49.0	37.6	96.5	33.3	63.0

In response to the query concerning additional remarks and recommendations regarding IPE, we received 34 responses. Of these, 21 participants indicated no further comments, while the remaining 13 responses provided valuable insights. They collectively acknowledged the educational value of the interprofessional collaboration intervention and voiced strong support for their continued integration, with no negative comments reported:

*‘Very enjoyable and highly interactive. This style of IPE should occur more frequently in a year and be repeated next year. A fantastic day’*. (428_student medicine)

Furthermore, participants consistently expressed a high degree of interest, characterizing the exercises as intellectually engaging and fostering effective learning. The interactive nature of the session and the ample opportunity for inquiries and collaboration were appreciated:

*‘It was very enjoyable to work with other disciplines; I learned a lot!’* (246_student nursing)

Respondents expressed a keen desire for continued involvement in such endeavors, emphasizing the advantages of interprofessional collaboration in knowledge acquisition. Collectively, these responses reflect the constructive impact of the IPE intervention, with healthcare students endorsing its continued integration into educational practices as a valuable and empowering learning experience.

## DISCUSSION

Our findings indicate that the IPE intervention had a positive and statistically significant impact on students’ competence, autonomy, and collaboration across midwifery, nursing, and medicine programs, aligning with the study objectives of evaluating shifts in students’ attitudes toward interprofessional collaboration and their perceptions of the intervention’s educational value and overall satisfaction. Students demonstrated improved confidence in their professional roles and a greater appreciation for interdisciplinary teamwork, reinforcing the effectiveness of structured IPE interventions. These findings emphasize the need for sustained integration of IPE in healthcare curricula to better prepare students for collaborative professional practice.

Our findings are consistent with existing literature, where IPE interventions are shown to improve students’ readiness for collaborative practice and enhance key professional competencies^5^. Students in our study gained valuable insights into each other’s strengths. This aligns with other studies demonstrating that interprofessional simulation exercises improve mutual understanding of professional roles^11^. Despite a slight decrease in perceived competence among medicine students’ post-intervention, their scores remained high overall, reflecting the intervention’s effectiveness in emphasizing the importance of interprofessional collaboration. Increased willingness to share information and resources, especially among midwifery and medicine students, further indicates improved collaboration. Similarly, nursing students placed a strong emphasis on teamwork, achieving the highest post-intervention score for collaborative work – echoing findings from Hallikainen et al.^12^, where students advocated for the inclusion of IPE in their curricula. Interestingly, our study found that the improvements in competence, autonomy, and collaboration were not significantly influenced by age, gender, or professional background.

Students’ positive feedback supports the integration of IPE into healthcare curricula, consistent with findings of Sabato et al.^13^ and Webster et al.^14^. Both our study and prior literature emphasize that IPE fosters effective collaboration, which is a key factor in improving patient care and professional development^15^. Our results underscore the need for expanding and systematizing IPE throughout healthcare education programs. Future research should focus on multi-step IPE interventions to explore their long-term impact on attitudes and competencies, ensuring comprehensive interprofessional development across healthcare professions^13^. Moreover, the creation of dedicated IPE learning pathways in healthcare programs would reinforce interprofessional collaboration and improve students’ clinical preparedness.

### Strengths and limitations

A key strength of this study is the structural integration of the interprofessional intervention into educational healthcare curricula, leading to actual interprofessional collaboration. Since 2021, annual interprofessional case walks through Brussels have been organized, involving midwifery, nursing, and medicine students. These walks offer experiential learning opportunities, exposing participants to diverse healthcare and welfare organizations while navigating the complexities of a metropolitan city like Brussels. While the Dutch version of the IEPS demonstrated reliability and good construct and convergent validity for the ‘Competence and autonomy’ and ‘Perception of actual cooperation’ subscales, the high correlation between these factors suggests potential issues with discriminant validity. Future research should include exploratory factor analysis to further investigate the dimensionality of the instrument and ensure that the subscales effectively measure distinct constructs^16^. Despite this, the intervention appears beneficial for students’ attitudes towards IPE. Some limitations warrant consideration. One limitation is the absence of a control group, which restricts the ability to definitively attribute observed changes to the intervention alone. Including a control group in future studies would improve the study’s internal validity and provide a stronger basis for causal inference. The high dropout rate – particularly among nursing students – raises concerns that the positive effects observed may be overestimated if those who withdrew did so because they did not benefit from or appreciated the intervention. The data exclusively reflect the attitudes of healthcare students within two specific settings in a single country, thus limiting generalizability. A more diverse sample might have produced different insights. Nevertheless, the principles and insights generated have the potential to contribute to advancements in healthcare education and collaboration internationally. Another limitation is the relatively high dropout rate after the post-measurement phase (42.8%), with notable attrition among nursing students (from 93.9% pre-test to 37.6% post-test). This remains unexplained, but variations in participation rates could indicate factors such as participant fatigue, time constraints, or changes in study conditions could have contributed to non-compliance or withdrawal from the study^17^.

## CONCLUSIONS

This study revealed a positive impact of an IPE intervention on competence, autonomy, and collaboration among healthcare students. A key strength of this study is its inclusion of three healthcare disciplines – midwifery, nursing, and medicine – allowing for a broader understanding of interprofessional collaboration beyond the commonly studied two-discipline models. Additionally, the use of simulation-based learning provided students with hands-on, scenario-driven experiences that closely reflect real-world clinical environments, further enhancing their readiness for interprofessional teamwork.

These findings not only align with existing research but also underscore the imperative for establishing dedicated IPE pathways and integrating interprofessional principles early into midwifery, nursing and medicine curricula. Future research should explore how the integration of IPE at different stages of education influences long-term professional collaboration and patient outcomes. Given the study’s quasi-experimental design and the absence of a control group, future research should incorporate randomized controlled trials to strengthen causal inferences regarding the effectiveness of IPE interventions. Moreover, additional studies could investigate the impact of IPE on clinical decision-making, patient-centered care, and interdisciplinary leadership skills.

By promoting effective collaboration between educators, practitioners, and researchers, this study contributes to the advancement of IPE methodologies and reinforces the importance of interprofessional education in strengthening healthcare systems globally.

## Supplementary Material



## Data Availability

The data supporting this research are available from the authors on reasonable request.
