# Supplementary material for: A quasi-experimental study on the impact of interprofessional education on collaborative attitudes among midwifery, nursing, and medicine students in Brussels, Belgium
Source: Eur J Midwifery. 2025 Jul 3;9:10.18332/ejm/204273. doi: 10.18332/ejm/204273 (PMC12224114; doi:10.18332/ejm/204273)
Supplement: Supplementary file 1 [file EJM-9-31-s1.pdf]

Reliability & Validity

April 19, 2023

Contents

0. About this document **3**

1 Data preparation **3**

2 Visual inspection **4**

3 The process of assessing reliability and validity **10**

3.1 Reliability scales based on previous publication 10

3.1.1 Competence & autonomy 10

3.1.2 Perceived need for cooperation 11

3.1.3 Perception of actual cooperation 11

3.1.4 Perception status own profession 12

3.2 Validity 13

3.2.1 How to read lavaan output? 13

3.2.2 The CFA models 14

3.2.2.1 The basic model 14

3.2.2.2 Basic model with DWLS estimator 16

3.2.2.3 Model with two dimensions 19

4 Final model: model with two dimensions **21**

4.1 Validity 21

4.2 Reliability final model 23

4.2.1 Scale 1: Competence and autonomy 23

4.2.2 Scale 2: Perception of actual cooperation 24

5 Appendix: relocate item 6 and 8 **26**
5.0.1 Model with two dimensions 26
5.0.2 Model with two dimensions, minus three items 28

0. About this document

This file contains a description of the analysis as requested. R code is displayed in gray boxes using this font. R output is displayed after double hashes. Notes and brief description of the code/results are shown in plain text. The analyses and corresponding results will be discussed in great detail with the researchers during an online consultation.

1 Data preparation

Load all needed R packages:

```
library(ggplot2)
library(foreign)
library(ggpubr) # for printing multiple ggplots
library(psych)
library(gridExtra) # for printing multiple ggplots
library(lavaan)
library(knitr) # for creating nice tables
```

Read in the data:

```
setwd("C:/Users/Lara/Documents/VUB/Consulting/2023/Analyses paper 2023")

df <- read.spss("IPO Dataset.sav", to.data.frame = T, use.value.labels = F)
rb <- df[,c(48:65)]
```

Check if the data type is correct for each variable:

```
str(rb)
```

```
## 'data.frame':    179 obs. of  18 variables:
##  $ VM1 : num  5 6 5 5 4 4 5 5 5 5 ...
##   ..- attr(*, "value.labels")= Named num [1:6] 6 5 4 3 2 1
##   .. ..- attr(*, "names")= chr [1:6] "Helemaal akkoord" "Matig akkoord" "Enigszins akkoord" "Enigszi
##  $ VM2 : num 5 6 5 5 5 5 5 5 4 5 ...
##   ..- attr(*, "value.labels")= Named num [1:6] 6 5 4 3 2 1
##   .. ..- attr(*, "names")= chr [1:6] "Helemaal akkoord" "Matig akkoord" "Enigszins akkoord" "Enigszi
##  $ VM3 : num 4 5 5 5 5 6 4 5 4 6 ...
##   ..- attr(*, "value.labels")= Named num [1:6] 6 5 4 3 2 1
##   .. ..- attr(*, "names")= chr [1:6] "Helemaal akkoord" "Matig akkoord" "Enigszins akkoord" "Enigszi
##  $ VM4 : num 6 6 5 4 5 3 5 6 4 6 ...
##   ..- attr(*, "value.labels")= Named num [1:6] 6 5 4 3 2 1
##   .. ..- attr(*, "names")= chr [1:6] "Helemaal akkoord" "Matig akkoord" "Enigszins akkoord" "Enigszi
##  $ VM5 : num 5 6 5 5 5 4 5 5 4 5 ...
##   ..- attr(*, "value.labels")= Named num [1:6] 6 5 4 3 2 1
##   .. ..- attr(*, "names")= chr [1:6] "Helemaal akkoord" "Matig akkoord" "Enigszins akkoord" "Enigszi
##  $ VM6 : num 6 6 6 6 5 6 6 6 4 6 ...
##   ..- attr(*, "value.labels")= Named num [1:6] 6 5 4 3 2 1
##   .. ..- attr(*, "names")= chr [1:6] "Helemaal akkoord" "Matig akkoord" "Enigszins akkoord" "Enigszi
##  $ VM7 : num 5 5 5 5 5 6 5 4 4 5 ...
##   ..- attr(*, "value.labels")= Named num [1:6] 6 5 4 3 2 1
```

```
##   .. ..- attr(*, "names")= chr [1:6] "Helemaal akkoord" "Matig akkoord" "Enigszins akkoord" "Enigszi
##  $ VM8 : num  6 6 6 6 5 3 5 6 4 5 ...
##   ..- attr(*, "value.labels")= Named num [1:6] 6 5 4 3 2 1
##   .. ..- attr(*, "names")= chr [1:6] "Helemaal akkoord" "Matig akkoord" "Enigszins akkoord" "Enigszi
##  $ VM9 : num  6 5 3 6 5 2 4 6 4 3 ...
##   ..- attr(*, "value.labels")= Named num [1:6] 6 5 4 3 2 1
##   .. ..- attr(*, "names")= chr [1:6] "Helemaal akkoord" "Matig akkoord" "Enigszins akkoord" "Enigszi
##  $ VM10: num  5 5 5 5 5 4 5 5 4 6 ...
##   ..- attr(*, "value.labels")= Named num [1:6] 6 5 4 3 2 1
##   .. ..- attr(*, "names")= chr [1:6] "Helemaal akkoord" "Matig akkoord" "Enigszins akkoord" "Enigszi
##  $ VM11: num  3 4 2 1 5 1 3 5 4 5 ...
##   ..- attr(*, "value.labels")= Named num [1:6] 6 5 4 3 2 1
##   .. ..- attr(*, "names")= chr [1:6] "Helemaal akkoord" "Matig akkoord" "Enigszins akkoord" "Enigszi
##  $ VM12: num  4 5 4 2 5 5 5 5 4 5 ...
##   ..- attr(*, "value.labels")= Named num [1:6] 6 5 4 3 2 1
##   .. ..- attr(*, "names")= chr [1:6] "Helemaal akkoord" "Matig akkoord" "Enigszins akkoord" "Enigszi
##  $ VM13: num  4 5 5 6 5 4 6 6 4 5 ...
##   ..- attr(*, "value.labels")= Named num [1:6] 6 5 4 3 2 1
##   .. ..- attr(*, "names")= chr [1:6] "Helemaal akkoord" "Matig akkoord" "Enigszins akkoord" "Enigszi
##  $ VM14: num  5 5 5 5 5 4 5 5 6 6 ...
##   ..- attr(*, "value.labels")= Named num [1:6] 6 5 4 3 2 1
##   .. ..- attr(*, "names")= chr [1:6] "Helemaal akkoord" "Matig akkoord" "Enigszins akkoord" "Enigszi
##  $ VM15: num  5 5 5 5 5 3 5 5 5 4 ...
##   ..- attr(*, "value.labels")= Named num [1:6] 6 5 4 3 2 1
##   .. ..- attr(*, "names")= chr [1:6] "Helemaal akkoord" "Matig akkoord" "Enigszins akkoord" "Enigszi
##  $ VM16: num  4 5 3 3 5 3 4 4 4 5 ...
##   ..- attr(*, "value.labels")= Named num [1:6] 6 5 4 3 2 1
##   .. ..- attr(*, "names")= chr [1:6] "Helemaal akkoord" "Matig akkoord" "Enigszins akkoord" "Enigszi
##  $ VM17: num  5 5 6 6 5 3 5 5 5 5 ...
##   ..- attr(*, "value.labels")= Named num [1:6] 6 5 4 3 2 1
##   .. ..- attr(*, "names")= chr [1:6] "Helemaal akkoord" "Matig akkoord" "Enigszins akkoord" "Enigszi
##  $ VM18: num  5 5 6 5 5 4 5 6 5 5 ...
##   ..- attr(*, "value.labels")= Named num [1:6] 6 5 4 3 2 1
##   .. ..- attr(*, "names")= chr [1:6] "Helemaal akkoord" "Matig akkoord" "Enigszins akkoord" "Enigszi
```

2 Visual inspection

Visual inspection of the distribution of the raw variables:

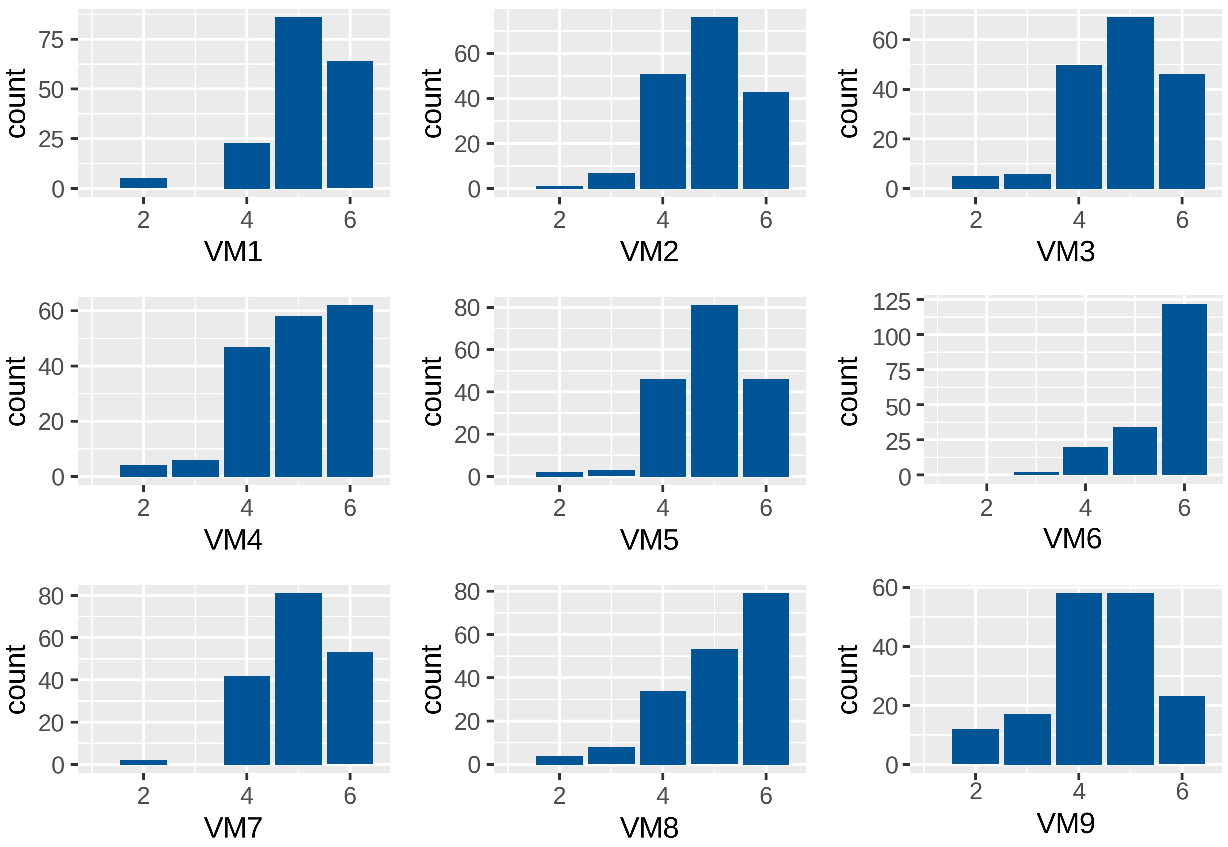
count
VM1
VM2
VM3
VM4
VM5
VM6
VM7
VM8
VM9

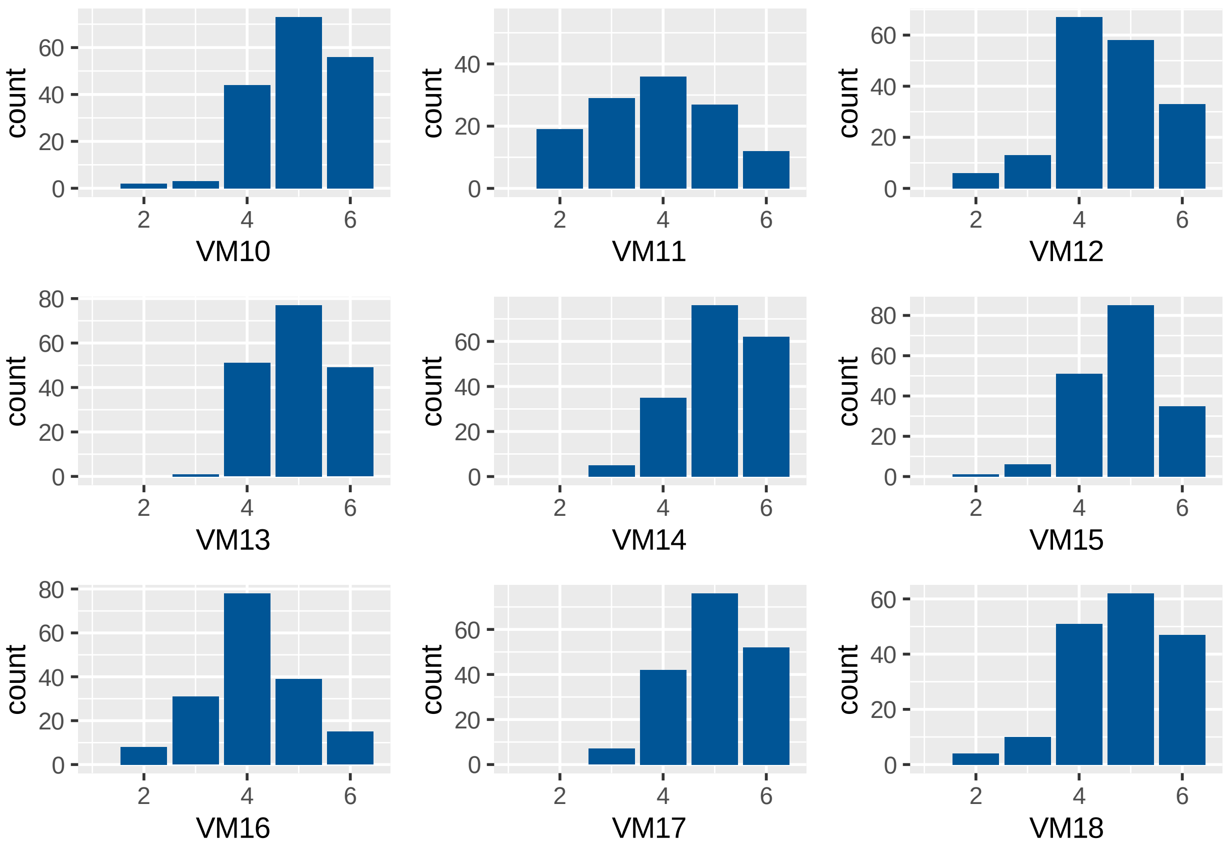

The value 1 is almost always absent. Request the tables:

VM1

Var1	Freq
2	5
4	23
5	86
6	64
NA	1

VM2

Var1	Freq
2	1
3	7
4	51
5	76
6	43
NA	1

VM3

Var1	Freq
1	2
2	5
3	6
4	50
5	69
6	46
NA	1

VM4

Var1	Freq
1	1
2	4
3	6
4	47
5	58
6	62
NA	1

VM5

Var1	Freq
2	2
3	3
4	46
5	81
6	46
NA	1

VM6

Var1	Freq
3	2
4	20
5	34
6	122
NA	1

VM7

Var1	Freq
2	2
4	42
5	81
6	53
NA	1

VM8

Var1	Freq
2	4
3	8
4	34
5	53
6	79
NA	1

VM9

Var1	Freq
1	10
2	12
3	17
4	58
5	58
6	23
NA	1

VM10

Var1	Freq
2	2
3	3
4	44
5	73
6	56
NA	1

VM11

Var1	Freq
1	55
2	19
3	29
4	36
5	27
6	12
NA	1

VM12

Var1	Freq
1	1
2	6
3	13
4	67
5	58
6	33
NA	1

VM13

Var1	Freq
3	1
4	51
5	77
6	49
NA	1

VM14

Var1	Freq
3	5
4	35
5	76
6	62
NA	1

VM15

Var1	Freq
2	1
3	6
4	51
5	85
6	35
NA	1

VM16

Var1	Freq
1	7
2	8
3	31
4	78
5	39
6	15
NA	1

VM17

Var1	Freq
3	7
4	42
5	76
6	52
NA	2

VM18

Var1	Freq
1	3
2	4
3	10
4	51
5	62
6	47
NA	2

3 The process of assessing reliability and validity

3.1 Reliability scales based on previous publication

The reliability of each scale is examined. For this purpose, Cronbach's alpha is used as a measure of internal consistency. Ideally, the alpha should be above .70 to be able to speak of an acceptable scale. From .80 onwards, one can speak of good internal consistency. When the scale only consists of two items, the Spearman-Brown coefficient is calculated (see below).

3.1.1 Competence & autonomy

```
##
## Reliability analysis
## Call: alpha(x = cbind(rb[, 1], rb[, 3], rb[, 4], rb[, 5], rb[, 7],
##     rb[, 9], rb[, 10], rb[, 13]))
##
##   raw_alpha std.alpha G6(smc) average_r S/N   ase mean   sd median_r
##        0.81      0.83    0.84      0.38   5 0.022  4.9 0.62      0.4
##
##     95% confidence boundaries
##          lower alpha upper
## Feldt     0.77  0.81  0.85
## Duhachek 0.77  0.81  0.85
##
##  Reliability if an item is dropped:
##     raw_alpha std.alpha G6(smc) average_r S/N alpha se  var.r med.r
## V1       0.79      0.81    0.82      0.39 4.4    0.025 0.0182  0.40
## V2       0.80      0.82    0.82      0.39 4.6    0.023 0.0136  0.41
## V3       0.78      0.81    0.81      0.38 4.3    0.025 0.0164  0.40
## V4       0.78      0.80    0.80      0.36 3.9    0.026 0.0108  0.38
## V5       0.78      0.80    0.80      0.37 4.1    0.025 0.0134  0.40
## V6       0.83      0.84    0.84      0.43 5.2    0.019 0.0093  0.43
## V7       0.79      0.81    0.82      0.38 4.3    0.025 0.0155  0.40
## V8       0.78      0.80    0.81      0.37 4.0    0.026 0.0153  0.38
##
##  Item statistics
##       n raw.r std.r r.cor r.drop mean   sd
## V1 178  0.66  0.67  0.59   0.55  5.1 0.85
## V2 178  0.63  0.63  0.56   0.48  4.8 1.03
## V3 178  0.70  0.69  0.65   0.58  4.9 1.02
## V4 178  0.74  0.76  0.75   0.65  4.9 0.83
## V5 178  0.71  0.74  0.71   0.62  5.0 0.80
## V6 178  0.57  0.50  0.39   0.35  4.2 1.30
```

```
## V7 178 0.67 0.68 0.62 0.55 5.0 0.86
## V8 178 0.72 0.75 0.70 0.63 5.0 0.77
##
## Non missing response frequency for each item
##         1    2    3    4    5    6 miss
## [1,] 0.00 0.03 0.00 0.13 0.48 0.36 0.01
## [2,] 0.01 0.03 0.03 0.28 0.39 0.26 0.01
## [3,] 0.01 0.02 0.03 0.26 0.33 0.35 0.01
## [4,] 0.00 0.01 0.02 0.26 0.46 0.26 0.01
## [5,] 0.00 0.01 0.00 0.24 0.46 0.30 0.01
## [6,] 0.06 0.07 0.10 0.33 0.33 0.13 0.01
## [7,] 0.00 0.01 0.02 0.25 0.41 0.31 0.01
## [8,] 0.00 0.00 0.01 0.29 0.43 0.28 0.01
```

We obtained a Cronbach's alpha of .81, which is good. This scale is internally consistent.

3.1.2 Perceived need for cooperation

This scale consists of only two items. In this case, Cronbach's alpha would often underestimate the true reliability. The Spearman-Brown coefficient, on the other hand, is on average less biased, especially if the correlation is very high. I recommend reading the following publication if more background on this topic is desired.

Eisinga, R., Grotenhuis, M. Te, & Pelzer, B. (2013). The reliability of a two-item scale: Pearson, Cronbach, or Spearman-Brown? *International Journal of Public Health, 58* (4), 637–642. https://doi.org/10.1007/s00038-012-0416-3

The spearman_brown() function from the splithalfr package calculates the Spearman-Brown prophecy formula for split-half reliability. This function requires complete cases and cannot handle missing values. Therefore, I need to remove the missing values before calculating the Spearman-Brown coefficient.

```
## [1] 0.5328001
```

The Spearman-Brown coefficient is a measure of the reliability of a test or scale. Specifically, it estimates what the reliability of the test or scale would be if it were lengthened or shortened. A higher Spearman-Brown coefficient indicates greater reliability, meaning that the test or scale is more consistent in measuring the construct of interest. A coefficient of 0.5 is generally considered a minimum acceptable level of reliability, although higher values are preferred.

However, note that the correlation between both items is relatively low (r = 0.36).

3.1.3 Perception of actual cooperation

```
##
## Reliability analysis
## Call: alpha(x = cbind(rb[, 2], rb[, 14], rb[, 15], rb[, 16], rb[, 17]))
##
##   raw_alpha std.alpha G6(smc) average_r S/N   ase mean   sd median_r
##        0.72      0.75    0.74      0.38   3 0.034  4.8 0.61     0.43
##
##    95% confidence boundaries
##          lower alpha upper
```

```
## Feldt       0.65   0.72   0.78
## Duhachek 0.66   0.72   0.79
##
##  Reliability if an item is dropped:
##     raw_alpha std.alpha G6(smc) average_r S/N alpha se   var.r  med.r
## V1      0.67      0.70    0.69      0.37 2.4    0.042 0.0428   0.36
## V2      0.67      0.71    0.70      0.38 2.4    0.042 0.0438   0.37
## V3      0.59      0.62    0.58      0.29 1.7    0.052 0.0265   0.31
## V4      0.80      0.80    0.77      0.51 4.1    0.024 0.0086   0.49
## V5      0.64      0.67    0.65      0.34 2.1    0.046 0.0349   0.38
##
##  Item statistics
##       n raw.r std.r  r.cor  r.drop  mean   sd
## V1 178  0.70  0.72   0.61   0.51   4.9 0.85
## V2 178  0.68  0.71   0.60   0.50   5.1 0.81
## V3 178  0.83  0.85   0.85   0.72   4.8 0.80
## V4 178  0.57  0.49   0.27   0.23   4.0 1.13
## V5 177  0.75  0.77   0.72   0.59   5.0 0.83
##
## Non missing response frequency for each item
##          1    2    3    4    5    6 miss
## [1,] 0.00 0.01 0.04 0.29 0.43 0.24 0.01
## [2,] 0.00 0.00 0.03 0.20 0.43 0.35 0.01
## [3,] 0.00 0.01 0.03 0.29 0.48 0.20 0.01
## [4,] 0.04 0.04 0.17 0.44 0.22 0.08 0.01
## [5,] 0.00 0.00 0.04 0.24 0.43 0.29 0.01
```

We find a Cronbach's alpha of .72, which is acceptable. If you were to remove item VM16 from the scale, you would obtain a Cronbach's alpha of 0.80.

3.1.4 Perception status own profession

```
##
## Reliability analysis
## Call: alpha(x = cbind(rb[, 11], rb[, 12], rb[, 18]))
##
##   raw_alpha std.alpha G6(smc) average_r S/N   ase mean   sd median_r
##        0.51      0.52    0.44      0.27 1.1 0.057  4.1 0.92     0.19
##
##      95% confidence boundaries
##           lower alpha upper
## Feldt       0.37   0.51   0.62
## Duhachek 0.40   0.51   0.63
##
##  Reliability if an item is dropped:
##     raw_alpha std.alpha G6(smc) average_r  S/N alpha se var.r med.r
## V1      0.29      0.29    0.17      0.17 0.42    0.105    NA  0.17
## V2      0.57      0.60    0.43      0.43 1.51    0.058    NA  0.43
## V3      0.29      0.32    0.19      0.19 0.47    0.094    NA  0.19
##
##  Item statistics
##       n raw.r std.r r.cor r.drop mean   sd
## V1 178  0.85  0.76  0.58   0.41  3.0  1.7
```

```
## V2 178  0.55  0.64  0.29   0.22  4.5  1.0
## V3 177  0.72  0.75  0.56   0.42  4.7  1.1
##
## Non missing response frequency for each item
##          1    2    3    4    5    6 miss
## [1,] 0.31 0.11 0.16 0.20 0.15 0.07 0.01
## [2,] 0.01 0.03 0.07 0.38 0.33 0.19 0.01
## [3,] 0.02 0.02 0.06 0.29 0.35 0.27 0.01
```

The reliability analysis shows that the Cronbach's alpha for the scale is 0.51, which is below the acceptable threshold of 0.70. This indicates that the scale may not be internally consistent. The G6(smc) value of 0.44 also indicates that there may be issues with item homogeneity.

Furthermore, the reliability analysis also shows that if item VM11 is dropped from the scale, the Cronbach's alpha will decrease even further to 0.29. This suggests that item V1 may be contributing positively to the reliability of the scale.

The scale cannot be used in its current form and removing an item is not a fruitful solution. For future research, I would suggest to consider adding more items to the scale or modifying the existing items to improve their homogeneity.

3.2 Validity

Next, I will perform some validity checks of the proposed scales. For this purpose, several CFA models are constructed. All models are constructed using the R package lavaan.

3.2.1 How to read lavaan output?

The output consists of three parts. The first six lines are called the header. The header contains the following information:

- the lavaan version number
- did lavaan converge normally or not, and how many iterations were needed
- the number of observations that were effectively used in the analysis
- the estimator that was used to obtain the parameter values
- the model test statistic, the degrees of freedom, and a corresponding p-value

The next section contains additional fit measures, and is only shown because we use the optional argument fit.measures = TRUE. It starts with the line `Model test baseline model` and ends with the value for the SRMR. These fit measures allow us to evaluate how well the proposed model fits the data. Stated differently, is the model adequate to explain the data?
First, if the chi-square test is non-significant, the model fit is considered acceptable since the observed covariance matrix is considered similar to the model-implied covariance matrix. The Comparative Fit Index (CFI) is suitable for small ($n < 100$) sample sizes (Bentler, 1989). It is advised that the CFI exceeds .90 or, ideally, .95. Next, a value of the Tucker Lewis Index (TLI) between .90 and .95 is considered as a marginal fit, values exceeding .95 represent a good fit (Kenny, 2014). Concerning the Root Mean-Square Error of Approximation (RMSEA) a value below 0.04 describes a good fit and below 0.08 a moderate fit (Kline, 2010).

The last section contains the parameter estimates. It starts with information about the standard errors (if the information matrix is expected or observed, and if the standard errors are standard, robust, or based on

the bootstrap). Then, it tabulates all free (and fixed) parameters that were included in the model. First the latent variables are shown, followed by covariances, intercepts, thresholds (due to the bound-argument) and (residual) variances. The first column Estimate contains the (estimated or fixed) parameter value for each model parameter; the second column Std.err contains the standard error for each estimated parameter; the third column Z-value contains the Wald statistic (which is simply obtained by dividing the parameter value by its standard error), the column P(>|z|) contains the p-value for testing the null hypothesis that the parameter equals zero in the population, and the last column Std.all contains the standardized parameter estimates (note that a standardized covariance is a correlation).
For the measurement model, we check if the standardized factor loadings are at least .50. *Note: parts of this clarifying text were simply copied from the lavaan website at https://lavaan.ugent.be/tutorial/cfa.html*

References

Bentler, P. M. (1989). EQS structural equations program manual. BMDP Statistical Software.
Cook, W. L. (1994). A Structural Equation Model of Dyadic Relationships within the Family System. Journal of Consulting and Clinical Psychology, 62(2), 500–509. https://doi.org/10.1037/0022-006X.62.3.500
Epskamp, S. (2015). semPlot: Unified Visualizations of Structural Equation Models. Structural Equation Modeling: A Multidisciplinary Journal, 22(3), 474–483. https://doi.org/10.1080/10705511.2014.937847
Kenny, D. A. (2014). Measuring Model Fit. http://davidakenny.net/cm/fit.htm
Kline, R. B. (2010). Principles and Practice of Structural Equation Modeling. The Guilford Press.
R Development Core Team. (2019). R: A Language and Environment for Statistical Computing. R Foundation for Statistical Computing. http://www.r-project.org/newline Rosseel, Y. (2012). Lavaan: An R Package for Structural Equation. Journal of Statistical Software, 48(2). www.jstatsoft.org

3.2.2 The CFA models

3.2.2.1 The basic model Note that the items are called VM followed by the number of the item in the questionnaire.

- The indicators for the latent variable 'Competence and autonomy' (ca) are: VM1, VM3, VM4, VM5, VM7, VM9, VM10 and VM13
- The indicators for the latent variable 'Percieved need for cooperation' (need) are: VM6 and VM8
- The indicators for the latent variable 'Perception of actual cooperation' (actual) are: VM2, VM14, VM15, VM16 and VM17

```
# Model 1: model met 3 dimensies
   mymodel <- '
    ca =~ VM1 + VM3 + VM4 + VM5 + VM7 + VM9 + VM10 + VM13
    need =~ VM6 + VM8
    actual =~ VM2 + VM14 + VM15 + VM16 + VM17
   '
fit <- cfa(mymodel, data = rb)
summary(fit, fit.measures = T, standardized = T)
```

```
## lavaan 0.6.14 ended normally after 49 iterations
##
##   Estimator                                         ML
##   Optimization method                           NLMINB
##   Number of model parameters                        33
##
##                                                   Used       Total
##   Number of observations                           177         179
```

```
##
## Model Test User Model:
##
##    Test statistic                              277.172
##    Degrees of freedom                               87
##    P-value (Chi-square)                          0.000
##
## Model Test Baseline Model:
##
##    Test statistic                             1107.843
##    Degrees of freedom                              105
##    P-value                                       0.000
##
## User Model versus Baseline Model:
##
##    Comparative Fit Index (CFI)                   0.810
##    Tucker-Lewis Index (TLI)                      0.771
##
## Loglikelihood and Information Criteria:
##
##   Loglikelihood user model (H0)            -3057.773
##   Loglikelihood unrestricted model (H1)    -2919.187
##
##    Akaike (AIC)                             6181.546
##    Bayesian (BIC)                           6286.359
##    Sample-size adjusted Bayesian (SABIC)    6181.854
##
## Root Mean Square Error of Approximation:
##
##    RMSEA                                         0.111
##    90 Percent confidence interval - lower        0.097
##    90 Percent confidence interval - upper        0.126
##    P-value H_0: RMSEA <= 0.050                   0.000
##    P-value H_0: RMSEA >= 0.080                   1.000
##
## Standardized Root Mean Square Residual:
##
##    SRMR                                          0.080
##
## Parameter Estimates:
##
##    Standard errors                            Standard
##    Information                                Expected
##    Information saturated (h1) model         Structured
##
## Latent Variables:
##                    Estimate  Std.Err  z-value  P(>|z|)   Std.lv  Std.all
##    ca =~
##      VM1             1.000                                0.465    0.548
##      VM3             1.216    0.211    5.771    0.000    0.566    0.548
##      VM4             1.259    0.211    5.960    0.000    0.586    0.575
##      VM5             1.403    0.194    7.217    0.000    0.653    0.789
##      VM7             1.269    0.182    6.973    0.000    0.590    0.739
##      VM9             0.881    0.238    3.708    0.000    0.410    0.316
```

```
##      VM10          1.164    0.183    6.347    0.000    0.542    0.632
##      VM13          1.206    0.174    6.936    0.000    0.561    0.732
##   need =~
##      VM6           1.000                               0.863    1.174
##      VM8           0.361    0.172    2.092    0.036    0.311    0.309
##   actual =~
##      VM2           1.000                               0.588    0.696
##      VM14          0.928    0.118    7.885    0.000    0.545    0.675
##      VM15          1.039    0.119    8.730    0.000    0.611    0.763
##      VM16          0.483    0.157    3.070    0.002    0.284    0.252
##      VM17          1.046    0.123    8.530    0.000    0.615    0.741
##
## Covariances:
##                  Estimate  Std.Err  z-value  P(>|z|)   Std.lv  Std.all
##   ca ~~
##      need          0.206    0.040    5.147    0.000    0.513    0.513
##      actual        0.214    0.042    5.075    0.000    0.783    0.783
##   need ~~
##      actual        0.181    0.041    4.426    0.000    0.356    0.356
##
## Variances:
##                  Estimate  Std.Err  z-value  P(>|z|)   Std.lv  Std.all
##    .VM1            0.504    0.057    8.891    0.000    0.504    0.699
##    .VM3            0.744    0.084    8.890    0.000    0.744    0.699
##    .VM4            0.697    0.079    8.814    0.000    0.697    0.670
##    .VM5            0.259    0.035    7.402    0.000    0.259    0.378
##    .VM7            0.289    0.036    7.943    0.000    0.289    0.453
##    .VM9            1.517    0.164    9.275    0.000    1.517    0.900
##    .VM10           0.441    0.051    8.605    0.000    0.441    0.601
##    .VM13           0.272    0.034    8.003    0.000    0.272    0.464
##    .VM6           -0.204    0.301   -0.679    0.497   -0.204   -0.378
##    .VM8            0.916    0.105    8.732    0.000    0.916    0.904
##    .VM2            0.367    0.047    7.781    0.000    0.367    0.515
##    .VM14           0.354    0.045    7.961    0.000    0.354    0.544
##    .VM15           0.268    0.038    6.985    0.000    0.268    0.418
##    .VM16           1.185    0.128    9.293    0.000    1.185    0.936
##    .VM17           0.311    0.043    7.295    0.000    0.311    0.451
##     ca             0.216    0.058    3.747    0.000    1.000    1.000
##     need           0.745    0.305    2.445    0.014    1.000    1.000
##     actual         0.346    0.070    4.927    0.000    1.000    1.000
```

This result in a bad model fit. Adapt the estimation technique to DWLS

```
fit2 <- cfa(mymodel, data = rb, estimator = "dwls")
summary(fit2, fit.measures = T, standardized = T)
```

3.2.2.2 Basic model with DWLS estimator

```
## lavaan 0.6.14 ended normally after 44 iterations
##
##   Estimator                                         DWLS
```

```
##   Optimization method                           NLMINB
##   Number of model parameters                        33
##
##                                                   Used       Total
##   Number of observations                           177         179
##
## Model Test User Model:
##
##   Test statistic                               100.284
##   Degrees of freedom                                87
##   P-value (Chi-square)                           0.156
##
## Model Test Baseline Model:
##
##   Test statistic                              1793.786
##   Degrees of freedom                               105
##   P-value                                        0.000
##
## User Model versus Baseline Model:
##
##   Comparative Fit Index (CFI)                    0.992
##   Tucker-Lewis Index (TLI)                       0.991
##
## Root Mean Square Error of Approximation:
##
##   RMSEA                                          0.029
##   90 Percent confidence interval - lower         0.000
##   90 Percent confidence interval - upper         0.053
##   P-value H_0: RMSEA <= 0.050                    0.924
##   P-value H_0: RMSEA >= 0.080                    0.000
##
## Standardized Root Mean Square Residual:
##
##   SRMR                                           0.076
##
## Parameter Estimates:
##
##   Standard errors                             Standard
##   Information                                 Expected
##   Information saturated (h1) model        Unstructured
##
## Latent Variables:
##                    Estimate  Std.Err  z-value  P(>|z|)   Std.lv  Std.all
##   ca =~
##     VM1               1.000                               0.462    0.543
##     VM3               1.110    0.119    9.326    0.000    0.513    0.496
##     VM4               1.245    0.125    9.952    0.000    0.576    0.563
##     VM5               1.398    0.124   11.320    0.000    0.647    0.779
##     VM7               1.245    0.119   10.441    0.000    0.576    0.719
##     VM9               0.856    0.128    6.691    0.000    0.396    0.304
##     VM10              1.174    0.115   10.192    0.000    0.543    0.632
##     VM13              1.287    0.113   11.435    0.000    0.595    0.775
##   need =~
##     VM6               1.000                               0.865    1.172
```

```
##       VM8                0.362    0.094    3.863    0.000    0.313    0.310
##    actual =~
##       VM2                1.000                               0.641    0.757
##       VM14               0.928    0.085   10.960    0.000    0.595    0.734
##       VM15               0.817    0.076   10.741    0.000    0.524    0.652
##       VM16               0.342    0.069    4.961    0.000    0.219    0.194
##       VM17               0.928    0.082   11.311    0.000    0.595    0.715
##
## Covariances:
##                       Estimate  Std.Err  z-value  P(>|z|)   Std.lv  Std.all
##    ca ~~
##       need               0.207    0.021    9.807    0.000    0.518    0.518
##       actual             0.240    0.022   10.678    0.000    0.809    0.809
##    need ~~
##       actual             0.180    0.029    6.120    0.000    0.325    0.325
##
## Variances:
##                       Estimate  Std.Err  z-value  P(>|z|)   Std.lv  Std.all
##     .VM1                 0.510    0.126    4.044    0.000    0.510    0.705
##     .VM3                 0.807    0.155    5.214    0.000    0.807    0.754
##     .VM4                 0.714    0.140    5.089    0.000    0.714    0.683
##     .VM5                 0.270    0.093    2.916    0.004    0.270    0.393
##     .VM7                 0.310    0.088    3.541    0.000    0.310    0.483
##     .VM9                 1.538    0.193    7.983    0.000    1.538    0.908
##     .VM10                0.444    0.092    4.851    0.000    0.444    0.601
##     .VM13                0.236    0.055    4.290    0.000    0.236    0.400
##     .VM6                -0.204    0.263   -0.775    0.438   -0.204   -0.374
##     .VM8                 0.921    0.124    7.453    0.000    0.921    0.904
##     .VM2                 0.306    0.088    3.482    0.000    0.306    0.427
##     .VM14                0.302    0.077    3.931    0.000    0.302    0.461
##     .VM15                0.371    0.078    4.739    0.000    0.371    0.575
##     .VM16                1.225    0.153    7.995    0.000    1.225    0.962
##     .VM17                0.339    0.076    4.471    0.000    0.339    0.489
##       ca                 0.214    0.030    7.138    0.000    1.000    1.000
##       need               0.748    0.253    2.954    0.003    1.000    1.000
##       actual             0.411    0.051    7.978    0.000    1.000    1.000
```

The model fit indices show a good model fit. However, we need to be cautious:
- VM6 negative variance (= Heywood case!)
- Problems with need scale, see std. factorloading of VM8
- VM16 not ok. This one lowered the Cronbach's alpha !
- VM9 not oke. Deleting this item, increased the Cronbach's alpha slightly.

During the consultation with client on the 18th of April 2023, we discussed the option to delete the scale "Perceived need for cooperation". In the Appendix, one can find the results when item 6 and 8 where moved to the 'Competence and autonomy' scale.

```
# Model 1: model met 3 dimensies
   model2dim <- '
    ca =~ VM1 + VM3 + VM4 + VM5 + VM7 + VM9 + VM10 + VM13
    actual =~ VM2 + VM14 + VM15 + VM16 + VM17
```

```
    '
fit2dim <- cfa(model2dim, data = rb)
summary(fit2dim, fit.measures = T, standardized = T)
```

3.2.2.3 Model with two dimensions

```
## lavaan 0.6.14 ended normally after 37 iterations
##
##   Estimator                                         ML
##   Optimization method                           NLMINB
##   Number of model parameters                        27
##
##                                                   Used       Total
##   Number of observations                           177         179
##
## Model Test User Model:
##
##   Test statistic                               232.353
##   Degrees of freedom                                64
##   P-value (Chi-square)                           0.000
##
## Model Test Baseline Model:
##
##   Test statistic                               971.351
##   Degrees of freedom                                78
##   P-value                                        0.000
##
## User Model versus Baseline Model:
##
##   Comparative Fit Index (CFI)                    0.812
##   Tucker-Lewis Index (TLI)                       0.770
##
## Loglikelihood and Information Criteria:
##
##   Loglikelihood user model (H0)              -2654.514
##   Loglikelihood unrestricted model (H1)      -2538.337
##
##   Akaike (AIC)                                5363.027
##   Bayesian (BIC)                              5448.784
##   Sample-size adjusted Bayesian (SABIC)       5363.279
##
## Root Mean Square Error of Approximation:
##
##   RMSEA                                          0.122
##   90 Percent confidence interval - lower         0.105
##   90 Percent confidence interval - upper         0.139
##   P-value H_0: RMSEA <= 0.050                    0.000
##   P-value H_0: RMSEA >= 0.080                    1.000
##
## Standardized Root Mean Square Residual:
##
##   SRMR                                           0.079
##
```

```
## Parameter Estimates:
##
##   Standard errors                             Standard
##   Information                                 Expected
##   Information saturated (h1) model          Structured
##
## Latent Variables:
##                    Estimate  Std.Err  z-value  P(>|z|)   Std.lv  Std.all
##   ca =~
##     VM1               1.000                               0.472    0.556
##     VM3               1.211    0.208    5.830    0.000    0.572    0.554
##     VM4               1.257    0.208    6.032    0.000    0.593    0.582
##     VM5               1.364    0.189    7.216    0.000    0.644    0.778
##     VM7               1.223    0.177    6.928    0.000    0.577    0.723
##     VM9               0.877    0.235    3.732    0.000    0.414    0.319
##     VM10              1.133    0.179    6.325    0.000    0.535    0.624
##     VM13              1.220    0.172    7.082    0.000    0.576    0.752
##   actual =~
##     VM2               1.000                               0.588    0.697
##     VM14              0.925    0.118    7.875    0.000    0.544    0.674
##     VM15              1.040    0.119    8.744    0.000    0.612    0.764
##     VM16              0.483    0.157    3.076    0.002    0.284    0.253
##     VM17              1.046    0.122    8.540    0.000    0.615    0.741
##
## Covariances:
##                    Estimate  Std.Err  z-value  P(>|z|)   Std.lv  Std.all
##   ca ~~
##     actual            0.218    0.043    5.106    0.000    0.785    0.785
##
## Variances:
##                    Estimate  Std.Err  z-value  P(>|z|)   Std.lv  Std.all
##    .VM1               0.497    0.056    8.810    0.000    0.497    0.691
##    .VM3               0.737    0.084    8.817    0.000    0.737    0.693
##    .VM4               0.688    0.079    8.725    0.000    0.688    0.661
##    .VM5               0.270    0.037    7.337    0.000    0.270    0.394
##    .VM7               0.304    0.038    7.935    0.000    0.304    0.477
##    .VM9               1.513    0.163    9.257    0.000    1.513    0.898
##    .VM10              0.448    0.052    8.554    0.000    0.448    0.610
##    .VM13              0.255    0.033    7.658    0.000    0.255    0.435
##    .VM2               0.367    0.047    7.783    0.000    0.367    0.515
##    .VM14              0.356    0.045    7.975    0.000    0.356    0.546
##    .VM15              0.267    0.038    6.978    0.000    0.267    0.417
##    .VM16              1.185    0.127    9.292    0.000    1.185    0.936
##    .VM17              0.310    0.043    7.293    0.000    0.310    0.451
##     ca                0.223    0.059    3.790    0.000    1.000    1.000
##     actual            0.346    0.070    4.930    0.000    1.000    1.000
```

Good model fit, no Heywood cases (e.g., negative variances). After inspecting the standardized factor loadings (see Std.all), we can conclude that the standardized factor loadings of item 9 and 16 are too low. Therefore, they are removed in the final model (see next section).

4 Final model: model with two dimensions

The final model contains two dimensions: "Competence & autonomy" (ca) and "Perception of actual cooperation" (need):
- Competence & autonomy: items 1, 3, 4, 5, 7, 10, and 13.
- Perception of actual cooperation: items 2, 14, 15, and 17.

4.1 Validity

```
# Model 1: model met 3 dimensies
    finalmodel <- '
      ca =~ VM1 + VM3 + VM4 + VM5 + VM7 + VM10 + VM13
      actual =~ VM2 + VM14 + VM15 + VM17
    '
fitfinal <- cfa(finalmodel, data = rb, estimator = "dwls")
summary(fitfinal, fit.measures = T, standardized = T)
```

```
## lavaan 0.6.14 ended normally after 31 iterations
##
##   Estimator                                         DWLS
##   Optimization method                             NLMINB
##   Number of model parameters                          23
##
##                                                     Used       Total
##   Number of observations                             177         179
##
## Model Test User Model:
##
##   Test statistic                                  46.087
##   Degrees of freedom                                  43
##   P-value (Chi-square)                             0.346
##
## Model Test Baseline Model:
##
##   Test statistic                                1427.279
##   Degrees of freedom                                  55
##   P-value                                          0.000
##
## User Model versus Baseline Model:
##
##   Comparative Fit Index (CFI)                      0.998
##   Tucker-Lewis Index (TLI)                         0.997
##
## Root Mean Square Error of Approximation:
##
##   RMSEA                                            0.020
##   90 Percent confidence interval - lower           0.000
##   90 Percent confidence interval - upper           0.056
##   P-value H_0: RMSEA <= 0.050                      0.902
##   P-value H_0: RMSEA >= 0.080                      0.001
##
```

```
## Standardized Root Mean Square Residual:
##
##   SRMR                                           0.071
##
## Parameter Estimates:
##
##   Standard errors                             Standard
##   Information                                 Expected
##   Information saturated (h1) model          Unstructured
##
## Latent Variables:
##                    Estimate  Std.Err  z-value  P(>|z|)   Std.lv  Std.all
##   ca =~
##     VM1               1.000                               0.451    0.530
##     VM3               1.131    0.131    8.604    0.000    0.510    0.493
##     VM4               1.237    0.136    9.108    0.000    0.558    0.545
##     VM5               1.427    0.136   10.513    0.000    0.643    0.775
##     VM7               1.260    0.131    9.653    0.000    0.568    0.709
##     VM10              1.186    0.126    9.439    0.000    0.535    0.622
##     VM13              1.351    0.127   10.649    0.000    0.609    0.792
##   actual =~
##     VM2               1.000                               0.644    0.761
##     VM14              0.910    0.086   10.648    0.000    0.586    0.724
##     VM15              0.814    0.077   10.522    0.000    0.524    0.653
##     VM17              0.907    0.082   11.010    0.000    0.584    0.702
##
## Covariances:
##                    Estimate  Std.Err  z-value  P(>|z|)   Std.lv  Std.all
##   ca ~~
##     actual            0.242    0.024   10.195    0.000    0.834    0.834
##
## Variances:
##                    Estimate  Std.Err  z-value  P(>|z|)   Std.lv  Std.all
##    .VM1               0.521    0.126    4.121    0.000    0.521    0.719
##    .VM3               0.810    0.156    5.205    0.000    0.810    0.757
##    .VM4               0.735    0.141    5.215    0.000    0.735    0.703
##    .VM5               0.274    0.094    2.920    0.003    0.274    0.399
##    .VM7               0.319    0.088    3.609    0.000    0.319    0.497
##    .VM10              0.453    0.092    4.913    0.000    0.453    0.613
##    .VM13              0.220    0.057    3.839    0.000    0.220    0.372
##    .VM2               0.302    0.089    3.396    0.001    0.302    0.421
##    .VM14              0.312    0.077    4.039    0.000    0.312    0.475
##    .VM15              0.370    0.079    4.704    0.000    0.370    0.574
##    .VM17              0.351    0.076    4.634    0.000    0.351    0.507
##     ca                0.203    0.031    6.599    0.000    1.000    1.000
##     actual            0.415    0.053    7.821    0.000    1.000    1.000
```

We now find an excellent model fit and all standardized factor loadings are high enough. Using this output, we can inspect three types of validity: construct validity, convergent validity and discriminant validity.

- Construct validity:
  The model appears to have good overall fit, as indicated by the comparative fit index (CFI) of 0.998 and Tucker-Lewis index (TLI) of 0.997. These indices assess how well the model fits the data compared to a baseline model with no factors or covariances estimated. The root mean square error

of approximation (RMSEA) is 0.020. The 90% confidence interval for RMSEA also does not include values above 0.080. This suggests that the model provides a good fit to the data. Additionally, the SRMR is 0.7

- Convergent validity:
  The factor loadings for the items on their respective factors are all significant and moderate to high, ranging from 0.493 to 0.775. This suggests that the factors are related to their respective items and that they are measuring the intended construct.

- Discriminant validity: The correlation between the latent factors ca and actual is 0.242, which is significant but moderate. This suggests that the factors are not measuring the same construct and provides some evidence of discriminant validity.

Overall, the model appears to have good construct, convergent and discriminant validity.

4.2 Reliability final model

4.2.1 Scale 1: Competence and autonomy

```
alpha(cbind(rb$VM1, rb$VM3,rb$VM4, rb$VM5, rb$VM7, rb$VM10,rb$VM13))
```

```
##
## Reliability analysis
## Call: alpha(x = cbind(rb$VM1, rb$VM3, rb$VM4, rb$VM5, rb$VM7, rb$VM10,
##     rb$VM13))
##
##   raw_alpha std.alpha G6(smc) average_r S/N   ase mean   sd median_r
##        0.83      0.84    0.84      0.43 5.2 0.019    5 0.62     0.43
##
##     95% confidence boundaries
##          lower alpha upper
## Feldt     0.79  0.83  0.87
## Duhachek 0.80  0.83  0.87
##
##  Reliability if an item is dropped:
##    raw_alpha std.alpha G6(smc) average_r S/N alpha se  var.r med.r
## V1      0.82      0.83    0.82      0.44 4.8    0.022 0.0116  0.46
## V2      0.82      0.83    0.82      0.45 4.8    0.020 0.0083  0.46
## V3      0.82      0.82    0.81      0.44 4.6    0.021 0.0093  0.43
## V4      0.79      0.80    0.79      0.40 4.0    0.024 0.0082  0.40
## V5      0.81      0.81    0.80      0.42 4.3    0.022 0.0072  0.43
## V6      0.81      0.82    0.82      0.44 4.6    0.022 0.0088  0.43
## V7      0.80      0.81    0.81      0.41 4.2    0.023 0.0110  0.43
##
##  Item statistics
##      n raw.r std.r r.cor r.drop mean   sd
## V1 178  0.67  0.67  0.58   0.54  5.1 0.85
## V2 178  0.68  0.66  0.58   0.52  4.8 1.03
## V3 178  0.70  0.69  0.63   0.55  4.9 1.02
## V4 178  0.79  0.80  0.77   0.71  4.9 0.83
```

```
##   V5 178    0.72     0.74     0.70       0.61     5.0  0.80
##   V6 178    0.68     0.69     0.62       0.55     5.0  0.86
##   V7 178    0.74     0.76     0.70       0.65     5.0  0.77
##
## Non missing response frequency for each item
##          1    2    3    4    5    6 miss
## [1,] 0.00 0.03 0.00 0.13 0.48 0.36 0.01
## [2,] 0.01 0.03 0.03 0.28 0.39 0.26 0.01
## [3,] 0.01 0.02 0.03 0.26 0.33 0.35 0.01
## [4,] 0.00 0.01 0.02 0.26 0.46 0.26 0.01
## [5,] 0.00 0.01 0.00 0.24 0.46 0.30 0.01
## [6,] 0.00 0.01 0.02 0.25 0.41 0.31 0.01
## [7,] 0.00 0.00 0.01 0.29 0.43 0.28 0.01
```

The reliability analysis indicates that the scale has high internal consistency, with a Cronbach's alpha coefficient of 0.83. The standard alpha and G6(smc) coefficients are also high, indicating good internal consistency. The average inter-item correlation is 0.43, and the S/N ratio is 5.2, indicating good signal to noise ratio.

The 95% confidence intervals for the Cronbach's alpha coefficient are between 0.79 and 0.87, indicating that the true reliability of the scale is likely to be within this range.

The reliability analysis also indicates that if any of the items were to be removed, the Cronbach's alpha coefficient would decrease, indicating that all items contribute to the internal consistency of the scale.

Overall, the results suggest that the scale has good internal consistency and all the items are contributing to the reliability of the scale.

4.2.2 Scale 2: Perception of actual cooperation

```
alpha(cbind(rb$VM2,  rb$VM14,rb$VM15,  rb$VM17))
```

```
##
## Reliability analysis
## Call: alpha(x = cbind(rb$VM2, rb$VM14, rb$VM15, rb$VM17))
##
##   raw_alpha std.alpha G6(smc) average_r S/N   ase mean   sd median_r
##         0.8       0.8    0.77      0.51 4.1 0.024  4.9 0.65     0.49
##
##    95% confidence boundaries
##          lower alpha upper
## Feldt     0.75   0.8  0.85
## Duhachek 0.76   0.8  0.85
##
## Reliability if an item is dropped:
##    raw_alpha std.alpha G6(smc) average_r S/N alpha se   var.r med.r
## V1      0.78      0.78    0.72      0.54 3.5    0.029 0.01602  0.52
## V2      0.78      0.78    0.73      0.55 3.6    0.029 0.01443  0.53
## V3      0.70      0.70    0.61      0.44 2.4    0.039 0.00024  0.44
## V4      0.75      0.75    0.67      0.50 3.0    0.032 0.00147  0.52
##
## Item statistics
##      n raw.r std.r r.cor r.drop mean   sd
```

```
## V1 178 0.77 0.76 0.63 0.57 4.9 0.85
## V2 178 0.75 0.76 0.62 0.56 5.1 0.81
## V3 178 0.85 0.86 0.82 0.72 4.8 0.80
## V4 177 0.80 0.80 0.72 0.63 5.0 0.83
##
## Non missing response frequency for each item
##         2    3    4    5    6 miss
## [1,] 0.01 0.04 0.29 0.43 0.24 0.01
## [2,] 0.00 0.03 0.20 0.43 0.35 0.01
## [3,] 0.01 0.03 0.29 0.48 0.20 0.01
## [4,] 0.00 0.04 0.24 0.43 0.29 0.01
```

The first table shows the results of the reliability analysis, where the raw alpha coefficient is 0.8, indicating a high level of internal consistency among the four items. The standard alpha coefficient is also 0.8, and the G6(smc) coefficient is 0.77, which indicates that the scale is measuring a single underlying construct. The average inter-item correlation (average_r) is 0.51, which is moderate, and the signal-to-noise ratio (S/N) is 4.1, indicating a good ratio of true score variance to error variance. The median inter-item correlation (median_r) is 0.49, and the standard error of measurement (ase) is 0.024. The mean score across the four items is 4.9, with a standard deviation (sd) of 0.65.

The next table shows the 95% confidence intervals for the alpha coefficient using two methods: Feldt and Duhachek. The confidence intervals are relatively narrow, indicating that the alpha coefficient is fairly precise.

The third table shows the reliability of the scale if each item is dropped. The raw alpha coefficients range from 0.70 to 0.78, indicating that all four items contribute to the internal consistency of the scale.

Overall, it can be concluded that this scale has good internal consistency.

5 Appendix: relocate item 6 and 8

In this appendix, items 6 and 8 were relocated to the 'Competence and autonomy' scale.

5.0.1 Model with two dimensions

Include VM6 and VM8 in the ca subscale

```
    mymodel2 <- '
     ca =~ VM1 + VM3 + VM4 + VM5 + VM7 + VM9 + VM10 + VM13 + VM6 + VM8
     actual =~ VM2 + VM14 + VM15 + VM16 + VM17
    '
fit3 <- cfa(mymodel2, data = rb, estimator = "dwls")
summary(fit3, fit.measures = T, standardized = T)
```

```
## lavaan 0.6.14 ended normally after 35 iterations
##
##   Estimator                                         DWLS
##   Optimization method                             NLMINB
##   Number of model parameters                          31
##
##                                                     Used       Total
##   Number of observations                             177         179
##
## Model Test User Model:
##
##   Test statistic                                 114.075
##   Degrees of freedom                                  89
##   P-value (Chi-square)                             0.038
##
## Model Test Baseline Model:
##
##   Test statistic                                1793.786
##   Degrees of freedom                                 105
##   P-value                                          0.000
##
## User Model versus Baseline Model:
##
##   Comparative Fit Index (CFI)                      0.985
##   Tucker-Lewis Index (TLI)                         0.982
##
## Root Mean Square Error of Approximation:
##
##   RMSEA                                            0.040
##   90 Percent confidence interval - lower           0.010
##   90 Percent confidence interval - upper           0.060
##   P-value H_0: RMSEA <= 0.050                      0.774
##   P-value H_0: RMSEA >= 0.080                      0.000
##
## Standardized Root Mean Square Residual:
##
##   SRMR                                             0.080
```

```
##
## Parameter Estimates:
##
##   Standard errors                             Standard
##   Information                                 Expected
##   Information saturated (h1) model          Unstructured
##
## Latent Variables:
##                    Estimate  Std.Err  z-value  P(>|z|)   Std.lv  Std.all
##   ca =~
##     VM1               1.000                               0.467    0.549
##     VM3               1.108    0.119    9.333    0.000    0.518    0.501
##     VM4               1.246    0.125    9.969    0.000    0.582    0.569
##     VM5               1.393    0.123   11.318    0.000    0.651    0.785
##     VM7               1.233    0.118   10.438    0.000    0.577    0.720
##     VM9               0.858    0.127    6.730    0.000    0.401    0.308
##     VM10              1.164    0.114   10.189    0.000    0.544    0.633
##     VM13              1.282    0.112   11.422    0.000    0.599    0.780
##     VM6               0.920    0.093    9.884    0.000    0.430    0.583
##     VM8               0.387    0.083    4.636    0.000    0.181    0.179
##   actual =~
##     VM2               1.000                               0.641    0.757
##     VM14              0.930    0.085   10.947    0.000    0.596    0.737
##     VM15              0.813    0.076   10.705    0.000    0.521    0.649
##     VM16              0.332    0.068    4.870    0.000    0.213    0.189
##     VM17              0.928    0.082   11.290    0.000    0.595    0.715
##
## Covariances:
##                    Estimate  Std.Err  z-value  P(>|z|)   Std.lv  Std.all
##   ca ~~
##     actual            0.236    0.022   10.678    0.000    0.788    0.788
##
## Variances:
##                    Estimate  Std.Err  z-value  P(>|z|)   Std.lv  Std.all
##    .VM1               0.506    0.126    4.003    0.000    0.506    0.698
##    .VM3               0.802    0.155    5.174    0.000    0.802    0.749
##    .VM4               0.707    0.141    5.025    0.000    0.707    0.676
##    .VM5               0.265    0.093    2.846    0.004    0.265    0.384
##    .VM7               0.309    0.088    3.525    0.000    0.309    0.482
##    .VM9               1.534    0.193    7.954    0.000    1.534    0.905
##    .VM10              0.442    0.092    4.828    0.000    0.442    0.599
##    .VM13              0.231    0.055    4.192    0.000    0.231    0.392
##    .VM6               0.359    0.076    4.736    0.000    0.359    0.660
##    .VM8               0.986    0.120    8.231    0.000    0.986    0.968
##    .VM2               0.306    0.088    3.472    0.001    0.306    0.427
##    .VM14              0.300    0.077    3.894    0.000    0.300    0.457
##    .VM15              0.373    0.078    4.774    0.000    0.373    0.579
##    .VM16              1.227    0.153    8.016    0.000    1.227    0.964
##    .VM17              0.338    0.076    4.458    0.000    0.338    0.488
##     ca                0.219    0.030    7.188    0.000    1.000    1.000
##     actual            0.411    0.052    7.963    0.000    1.000    1.000
```

```
anova(fit2, fit3)
```

```
##
## Chi-Squared Difference Test
##
##      Df AIC BIC  Chisq Chisq diff    RMSEA Df diff Pr(>Chisq)
## fit2 87          100.28
## fit3 89          114.08     13.791 0.18251       2   0.001012 **
## ---
## Signif. codes:  0 '***' 0.001 '**' 0.01 '*' 0.05 '.' 0.1 ' ' 1
```

```
MI <- modindices(fit3)
sortedModInd <- MI[order(-MI$mi),] ; sortedModInd[1:10,]
```

```
##        lhs op  rhs     mi    epc sepc.lv sepc.all sepc.nox
## 36      ca =~ VM15 13.429 -1.371  -0.641   -0.798   -0.798
## 133    VM6 ~~  VM8 11.527  0.208   0.208    0.350    0.350
## 152   VM15 ~~ VM17  9.305  0.196   0.196    0.552    0.552
## 112    VM9 ~~  VM8  8.177  0.287   0.287    0.233    0.233
## 44  actual =~  VM9  7.453 -1.065  -0.683   -0.525   -0.525
## 46  actual =~ VM13  7.397  0.626   0.401    0.522    0.522
## 48  actual =~  VM8  7.060 -0.726  -0.465   -0.461   -0.461
## 132   VM13 ~~ VM17  5.835  0.124   0.124    0.444    0.444
## 77     VM4 ~~  VM7  4.722 -0.154  -0.154   -0.329   -0.329
## 151   VM15 ~~ VM16  4.307  0.175   0.175    0.259    0.259
```

5.0.2 Model with two dimensions, minus three items

Delete 3 items (VM8, VM9, VM16):

```
mymodel3 <- '
    ca =~ VM1 + VM3 + VM4 + VM5 + VM7 + VM10 + VM13 + VM6
    actual =~ VM2 + VM14 + VM15 + VM17
   '
fit4 <- cfa(mymodel3, data = rb, estimator = "dwls")
summary(fit4, fit.measures = T, standardized = T)
```

```
## lavaan 0.6.14 ended normally after 32 iterations
##
##   Estimator                                         DWLS
##   Optimization method                             NLMINB
##   Number of model parameters                          25
##
##                                                     Used       Total
##   Number of observations                             177         179
##
```

```
## Model Test User Model:
##
##   Test statistic                               52.420
##   Degrees of freedom                               53
##   P-value (Chi-square)                          0.497
##
## Model Test Baseline Model:
##
##   Test statistic                             1628.674
##   Degrees of freedom                               66
##   P-value                                       0.000
##
## User Model versus Baseline Model:
##
##   Comparative Fit Index (CFI)                   1.000
##   Tucker-Lewis Index (TLI)                      1.000
##
## Root Mean Square Error of Approximation:
##
##   RMSEA                                         0.000
##   90 Percent confidence interval - lower        0.000
##   90 Percent confidence interval - upper        0.047
##   P-value H_0: RMSEA <= 0.050                   0.967
##   P-value H_0: RMSEA >= 0.080                   0.000
##
## Standardized Root Mean Square Residual:
##
##   SRMR                                          0.069
##
## Parameter Estimates:
##
##   Standard errors                            Standard
##   Information                                Expected
##   Information saturated (h1) model        Unstructured
##
## Latent Variables:
##                    Estimate  Std.Err  z-value  P(>|z|)   Std.lv  Std.all
##   ca =~
##     VM1               1.000                               0.453    0.532
##     VM3               1.134    0.125    9.062    0.000    0.513    0.496
##     VM4               1.246    0.130    9.604    0.000    0.564    0.552
##     VM5               1.439    0.131   11.012    0.000    0.651    0.785
##     VM7               1.276    0.126   10.162    0.000    0.578    0.721
##     VM10              1.199    0.121    9.924    0.000    0.543    0.631
##     VM13              1.319    0.119   11.115    0.000    0.597    0.777
##     VM6               0.932    0.098    9.527    0.000    0.422    0.572
##   actual =~
##     VM2               1.000                               0.644    0.761
##     VM14              0.926    0.084   10.959    0.000    0.596    0.736
##     VM15              0.796    0.074   10.700    0.000    0.512    0.638
##     VM17              0.908    0.080   11.288    0.000    0.584    0.702
##
## Covariances:
##                    Estimate  Std.Err  z-value  P(>|z|)   Std.lv  Std.all
```

```
##    ca ~~
##      actual            0.241    0.023   10.535    0.000    0.826    0.826
##
## Variances:
##                   Estimate  Std.Err  z-value  P(>|z|)   Std.lv  Std.all
##     .VM1             0.519    0.126    4.117    0.000    0.519    0.717
##     .VM3             0.807    0.155    5.202    0.000    0.807    0.754
##     .VM4             0.727    0.140    5.187    0.000    0.727    0.696
##     .VM5             0.264    0.093    2.830    0.005    0.264    0.384
##     .VM7             0.308    0.088    3.497    0.000    0.308    0.480
##     .VM10            0.444    0.092    4.839    0.000    0.444    0.601
##     .VM13            0.234    0.055    4.223    0.000    0.234    0.396
##     .VM6             0.366    0.076    4.830    0.000    0.366    0.673
##     .VM2             0.302    0.088    3.418    0.001    0.302    0.422
##     .VM14            0.300    0.077    3.891    0.000    0.300    0.458
##     .VM15            0.383    0.078    4.915    0.000    0.383    0.593
##     .VM17            0.351    0.075    4.662    0.000    0.351    0.507
##      ca              0.205    0.030    6.924    0.000    1.000    1.000
##      actual          0.415    0.052    7.937    0.000    1.000    1.000
```

```
anova(fit3, fit4)
```

```
##
## Chi-Squared Difference Test
##
##      Df AIC BIC  Chisq Chisq diff     RMSEA Df diff Pr(>Chisq)
## fit4 53             52.42
## fit3 89            114.08     61.655 0.063452      36   0.004912 **
## ---
## Signif. codes:  0 '***' 0.001 '**' 0.01 '*' 0.05 '.' 0.1 ' ' 1
```

```
  # significant betere modelfit (opgelet: niet dezelfde geobserveerde variabelen!). AIC niet beschikba
```

Appendix 2: Validated Dutch version of the IEPS questionnaire

**Subscale: Competence and autonomy**
People from my professional group demonstrate great autonomy.
People from other professional groups respect the work performed by my professional group.
People from my professional group have a very positive attitude toward their own goals and objectives.
People from my professional group need to collaborate with other professional groups.
People from my professional group are very positive about their contributions and achievements.
People from my professional group trust each other's professional judgment.
People from my professional group are highly competent.
**Subscale: Perception of actual cooperation**
People from my professional group are capable of collaborating closely with people from other professional groups.
People from my professional group are willing to share information and resources with other professional groups.
People from my professional group have good relationships with people in other professional groups.
People from my professional group work well together.

© 2025 Vermeulen J. et al.
